# Criminal clickbait: a panel data analysis on the attractiveness of online advertisements offering stolen data

**DOI:** 10.3389/fdata.2023.1320569

**Published:** 2023-12-22

**Authors:** Renushka Madarie, Christianne de Poot, Marleen Weulen Kranenbarg

**Affiliations:** ^1^Research Group Forensic Science, Faculty of Technology, Amsterdam University of Applied Sciences, Amsterdam, Netherlands; ^2^Department of Criminology, Faculty of Law, Vrije Universiteit (VU) Amsterdam, Amsterdam, Netherlands; ^3^Police Academy of the Netherlands, Apeldoorn, Netherlands

**Keywords:** stolen account credentials, darkweb markets, cybercrime, illegal trade, reputation, rational choice, consumer behaviour

## Abstract

**Introduction:**

Few studies have examined the sales of stolen account credentials on darkweb markets. In this study, we tested how advertisement characteristics affect the popularity of illicit online advertisements offering account credentials. Unlike previous criminological research, we take a novel approach by assessing the applicability of knowledge on regular consumer behaviours instead of theories explaining offender behaviour.

**Methods:**

We scraped 1,565 unique advertisements offering credentials on a darkweb market. We used this panel data set to predict the simultaneous effects of the asking price, endorsement cues and title elements on advertisement popularity by estimating several hybrid panel data models.

**Results:**

Most of our findings disconfirm our hypotheses. Asking price did not affect advertisement popularity. Endorsement cues, including vendor reputation and cumulative sales and views, had mixed and negative relationships, respectively, with advertisement popularity.

**Discussion:**

Our results might suggest that account credentials are not simply regular products, but high-risk commodities that, paradoxically, become less attractive as they gain popularity. This study highlights the necessity of a deeper understanding of illicit online market dynamics to improve theories on illicit consumer behaviours and assist cybersecurity experts in disrupting criminal business models more effectively. We propose several avenues for future experimental research to gain further insights into these illicit processes.

## 1 Introduction

Stolen personal data is of increasing importance to crimes committed online (Porcedda and Wall, 2021). Stolen account credentials in particular are often not only the end result of an online attack such as hacking, but are also used as stepping stones to other types of crime (Onaolapo et al., 2016; Thomas et al., 2017; Nathan, 2020). The attractiveness of this type of stolen data lies in its versatility (Shulman, 2010). For instance, offenders could monetise credentials by stealing money from stolen bank or webshop accounts, or by using them to defraud contacts of the account holder (Shay et al., 2014). Stolen credentials are disseminated in vast amounts as evidenced by websites selling stolen credentials (e.g., weleakinfo.com) and websites alerting individuals about their leaked credentials (e.g., haveibeenpwned.com or scatteredsecrets.com). Online platforms enabling the illicit dissemination of credentials not only promote account abuse, but also create an additional incentive to steal credentials by facilitating the sale of this data.

A wide range of online platforms enable the sale of stolen account credentials, varying from mobile chat applications to hacker forums and illicit online marketplaces on the web (Madarie et al., 2019; Hebel et al., 2021). Typical illicit online markets are so-called “darkweb” markets that can only be reached with anonymisation software such as the Tor browser. This software facilitates technical anonymity by, amongst others, hiding the physical locations of market users and webservers hosting the market (Tor Project, 2023). When discussing illicit online markets in this paper, we refer to these darkweb markets. These markets come in different shapes and sizes. Some are specialised in, for example, the sales of drugs. Other markets provide a platform to offer all sorts of illicit products and services, such as counterfeits, hacking tutorials, and stolen account credentials (Ablon et al., 2014; Dolliver, 2015). While vendors and buyers attempt to maintain full anonymity, the marketplaces are generally of a global and open nature which makes them highly intriguing for research.

The open nature of several darkweb allows potential customers to find, access, and explore the market relatively easily. The global nature of the internet combined with the sales of illicit products desired by offenders across the world fosters illicit markets with an international customer base (Ablon et al., 2014; Duxbury and Haynie, 2018). Although this larger reach of markets increases the pool of potential customers, it also attracts more vendors and thus more sales competition. In order to understand who is more successful on these markets and why, we aim to analyse the effectiveness of several sales tactics used by illicit vendors selling account credentials to attract buyers. By doing so, this empirical paper advances theoretical insights into cybercrime and criminal decision-making and provides guidance to improve law enforcement interventions directed at disrupting market dynamics on illicit online markets.

Instead of testing traditional criminological theories, we adopt a novel approach by departing from general knowledge on consumer behaviour to study criminal consumption. We argue that criminal consumer behaviour is likely similar to consumer behaviour exhibited on licit online marketplaces. In both cases, consumers intend to trade. They offer money and expect a product with certain qualities in return. Furthermore, in both cases, the decision to trade involves a degree of uncertainty for the consumer. This uncertainty stems partly from information asymmetry which implies that only the seller has full information about the quality of the product on offer (Herley and Florêncio, 2010). In addition, compared to offline markets, it is relatively hard to verify the selling party and their intentions on the internet. To make an assessment about the quality of the product and trustworthiness of the vendor, consumers of both licit and illicit online markets have to rely on online cues, such as reputation ratings and the number of previous sales (Herley and Florêncio, 2010; Metzger and Flanagin, 2013; Norbutas et al., 2020). Illicit online markets generally apply similar reputation mechanisms as their licit counterparts (Haslebacher et al., 2017). We thus expect similar online cues to affect licit and illicit online consumer behaviour.

Few previous studies on illicit online markets explicitly depart from theories relating to licit consumer behaviour and traditional (offline) market dynamics. Some notable exceptions are Paquet-Clouston et al. (2018) who examined the structural features of online drugs markets based on features of offline illicit markets, and Howell et al. (2023) who deployed ecological and economic principles to analyse a number of darkweb markets. Furthermore, Norbutas et al. (2020) noted that trust problems apply to both illicit and licit online markets. In addition, Ablon et al. (2014) also briefly state that illicit markets are subject to the same microeconomic fluctuations of supply and demand as traditional markets.

Furthermore, despite the sizeable body of literature on darkweb markets and market dynamics, most studies focused on drugs trade (e.g., Hardy and Norgaard, 2016; Nurmi et al., 2017; Tsuchiya and Hiramoto, 2021) or on specific aspects of online trade, such as trust mechanisms or risk avoidance behaviours (Yip et al., 2013; Dupont et al., 2017; Norbutas et al., 2020; Howell et al., 2022), or prices (e.g., Holt et al., 2016; Steel, 2019). Moreover, the large majority of studies on darkweb markets appears to be descriptive (e.g., Hutchings and Holt, 2015; Haslebacher et al., 2017; Copeland et al., 2020).

By considering knowledge on licit consumer behaviours, we aim to expand our view on the factors affecting decision-making on illicit online markets. Moreover, by taking multiple factors into account and by using both longitudinal and cross-sectional data (i.e., panel data), we are able to compare their relative importance. To the best of our knowledge, no prior study has provided a more comprehensive quantitative assessment of different factors that could influence criminal consumers' decision-making on illicit online markets. To create more insight in this decision-making process, we examine three types of factors relevant to general consumer behaviour, namely (1) the asking price, (2) endorsement cues, and (3) title appearance. To assess their simultaneous effect on advertisement popularity, we used longitudinal data that we scraped from a darkweb market for a period of two months.

## 2 Theoretical framework and hypotheses

### 2.1 Consumer and criminal decision-making

Regular modern-day consumers looking to buy a product can usually choose from a variety of alternatives offered on the internet. However, not all alternatives are given equal thought and some alternatives are not considered at all (Narayana and Markin, 1975). In the past, consumer preferences were mainly studied through the lens of the utilitarian perspective (Sheth et al., 1991; Smith and Rupp, 2003). This perspective assumes that individuals act rationally to maximise their benefits and minimise their costs. For instance, if a consumer finds two products that are similar in all respects except price, the utilitarian perspective would assume the cheaper version to be chosen.

However, consumer decision-making is known to be affected by several limitations (Mathis and Steffen, 2015) which results in bounded rationality (Simon, 1972; Arthur, 1994). For instance, consumers do not have infinite amounts of time to arrive at optimal decisions. Furthermore, humans have only a limited cognitive capacity for information processing (Simon, 1972). To cope with these external and internal restrictions, consumers rely on heuristics, or mental shortcuts (Metzger and Flanagin, 2013). Heuristics involve information processing strategies that reduce cognitive load which in turn increases the speed and reduces the effort of decision-making. Shortcuts frequently used in the context of shopping encompass, for instance, reliance on product signals or cues that communicate underlying qualities of a product (Wernerfelt, 1988; Solomon et al., 2014). Examples of such cues are brand names and feedback from previous customers (Tsao et al., 2011; Kim, 2021).

Situational and contextual factors could also affect consumer decision-making. For example, more thought is put into the evaluation of alternatives when there is a perceived risk, which is the case with unfamiliar products, brands, and vendors (Solomon et al., 2014). Darkweb markets could be considered as particularly risky environments. Not only is there the possibility of being ripped off, buyers can also not turn to formal protective institutions, such as regulatory bodies and insurance companies (Herley and Florêncio, 2010; Lusthaus, 2012).

The utilitarian cost-benefit analysis that consumers adopt when shopping online, including the consideration of risk, has also been examined in studies on criminal decision-making. Most notably, Clarke and Cornish (1985) proposed the use of a rational choice framework to analyse criminal behaviour. This framework assumes that offenders aim to maximise their rewards while minimising the effort and risk when committing a crime (Loughran et al., 2016; Cornish and Clarke, 2017). Just as the rational choice framework has been embraced by a range of disciplines, so has it been applied to study a range of crimes (Loughran et al., 2016), varying from sexual crimes (Beauregard et al., 2010), burglary (Vandeviver and Bernasco, 2020) and money laundering (Gilmour, 2016) to online stolen data markets (Smirnova and Holt, 2017; Madarie et al., 2019).

In addition to utilitarian considerations, sales tactics are also aimed at attracting attention in the first place (Pieters and Wedel, 2004). In settings with multiple vendors, each vendor has to compete for the attention of the consumer. A range of tactics exist to draw consumers' attention. In print advertisements, this can be done with, amongst others, graphics and text (Pieters and Wedel, 2004). For instance, advertisers could vary the graphical design of their advertisements (Pieters et al., 2010), use images to draw attention to specific parts of an advertisement (Hutton and Nolte, 2011), or write arousing titles to sway people to read further (Subotic and Mukherjee, 2014; Pengnate, 2019).

In this study, we aim to better understand the rational decision-making process as well as to what extent criminal consumers are affected by sales tactics aimed at attracting attention. To this end, we examine several advertisement characteristics that might affect the popularity of advertisements offering account credentials on an illicit online market. Advertisements are listed on overview pages that provide basic information on the advertisements, such as the price and rating of the vendor along with the listing title. Clicking on an advertisement loads the full advertisement page that details, for instance, more information about the product and additional terms of service. This clicking signals interest from a potential buyer. Some advertisements are clicked on more often than others. What drives these varying levels of interest? To answer this research question, we assess the simultaneous effect of several key advertisement characteristics on advertisement popularity in an illicit setting. More specifically, we examine the effects of asking price, endorsement cues, and title appearance on the number of clicks (i.e., *view* counts) on an advertisement.

### 2.2 Asking price

Advertisements on licit and illicit online markets often prominently display the asking price for the offered product, as prices are well-known to affect consumer's decision-making process (Niedrich et al., 2001; Lalwani and Monroe, 2005). From a utilitarian perspective, similar products with lower prices would be more attractive because lower costs imply a higher net gain in the utility function. When factoring in increased transaction risks due to rippers (i.e., sellers who claim to sell something, but intentionally do not deliver as promised) and a lack of measures protecting consumers' rights on illicit markets, lower prices could make products even more attractive (Agarwal and Teas, 2001). Therefore, consumers might opt to buy a cheaper version of the same product in order to minimise any potential (financial) loss.

However, there are reasons why consumers would want to pay a higher price. For instance, it has been argued that higher prices signal higher product quality (Wolinsky, 1983; Anderson and Simester, 2003). Higher quality products might be costlier to make and this higher cost would be passed on to consumers. Conversely, discounted items could be perceived as defects or leftovers. More expensive products could thus be perceived as more valuable and, therefore, more desirable (Zeithaml, 1988; Anderson and Simester, 2003).

Similarly, consumers on illicit online markets might use heuristics to process the large number of advertisements on illicit online markets (Solomon et al., 2014). A thorough processing of advertisements might be hindered due to limitations of our cognitive capacity and the riskiness inherent to this illicit environment. In this context, research suggests that price would be used as a quality measure rather than a cost measure (Suri et al., 2007). In other words, buyers would perceive a higher price to signal a higher value rather than as a monetary sacrifice.

Conversely, when consumers are motivated and able to systematically process information, price is more likely to be evaluated as a cost measure (Suri et al., 2007). In line with this reasoning, research on online drugs trade demonstrated that higher product prices relate to less sales (Przepiorka et al., 2017; Norbutas et al., 2020). This suggests that buyers of these illicit products indeed use price as a measure of financial cost in transactions. To date, research on stolen data pricings only reported descriptive results (e.g., Hutchings and Holt, 2015; Haslebacher et al., 2017). Hence, it is uncertain how prices affect potential buyers of stolen data. Consequently, based on general consumer literature, we hypothesise that the asking price affects the attractiveness of advertisements offering credentials, but we do not speculate on the direction of this effect.

*H1: The asking price relates to view count increases*.

### 2.3 Endorsement cues

Whereas, paying for a product online is relatively straightforward, what happens after payment is not always certain for buyers. Although nearly all online markets are characterised by information asymmetry, this problem is exacerbated on illicit online markets (Herley and Florêncio, 2010; Norbutas et al., 2020). In general, only vendors know the true quality of their products, whereas buyers can only guess. Furthermore, vendors have an incentive to highlight positive qualities of a product and conceal its negative qualities which fuels low trust in vendors. Moreover, vendors operate in anonymity on illicit online markets. Anonymity is encouraged to decrease the risk of apprehension by law enforcement and enhanced by technical anonymisation techniques (Lusthaus, 2012). Moreover, if buyers get ripped off, they cannot fall back on formal protection agencies such as the police or insurance companies due to the illicit nature of the transaction. These conditions result in a high-risk environment for buyers which could negatively affect purchase intentions (Bianchi and Andrews, 2012).

To overcome this trust problem, darkweb markets, like most other online markets, install measures to reduce this uncertainty (and increase trust), such as vendor and product rating systems (Holt and Lampke, 2010; Yip et al., 2013; Haslebacher et al., 2017; Przepiorka et al., 2017). These measures hinge on consumers using heuristics that involve judgements based on other people's behaviour (Sundar, 2008; Metzger and Flanagin, 2013). More specifically, research on licit consumer behaviours demonstrated that if other people show endorsement of a product or vendor, for instance through reviews or ratings, buyers are inclined to believe that they themselves would also like the product or trust the vendor (Serra Cantallops and Salvi, 2014; Kim et al., 2019). This increased trust could subsequently translate to a higher likelihood of purchasing.

A large number of studies has demonstrated that our decision-making is positively affected by online reviews and ratings of persons or products (e.g., Dellarocas, 2003; Chevalier and Mayzlin, 2006; Salganik et al., 2006; van de Rijt et al., 2014). On illicit online markets, this is no different. Vendors could establish their reputation on these markets in generally two ways: either they become verified, for instance by providing a sample of their goods to the market administrators, or they earn their status by obtaining (positive) feedback from previous customers (Holt and Lampke, 2010; Hutchings and Holt, 2015; Dupont et al., 2016). Vendors' rating could thus refer to the quality of their products and services (Yip et al., 2013; Ablon et al., 2014). Studies quantifying the relationship between vendors' reputation and illicit drugs sales demonstrated that a higher reputation yields more sales (Nurmi et al., 2017; Przepiorka et al., 2017; Norbutas et al., 2020). Therefore, we also expect to find a positive effect of a vendors' reputation on the attractiveness of advertisements offering credentials.

*H2: Vendor rankings positively relate to view count increases*.

In addition, earlier clicks on the advertisement could also imply that other potential buyers believed the advertised product to be interesting. In a similar vein, past sales of the product advertised could imply that previous buyers believed the vendor to be trustworthy. Therefore, we expect that the number of cumulative clicks (i.e., how many views the advertisement had already generated) and sales affect the attractiveness of an advertisement as well. This is in line with Norbutas et al. (2020) demonstrating that the accumulated number of sales positively affects subsequent sales of drugs.

*H3: The cumulative view count positively relates to view count increases*.

*H4: The number of previous sales positively relates to view count increases*.

### 2.4 Title characteristics

While previous studies examining darkweb markets dynamics have included price and vendor status in their analyses, we know of no study that specifically considered title characteristics of illicit advertisement as well. However, from a consumer perspective, it makes sense to examine the effects of title characteristics on decision-making. Because consumers do not have infinite amounts of time, they will likely not thoroughly evaluate all advertisements on overview pages. For an advertisement to be evaluated, it has to receive attention first. One of the advertisement attributes purposefully tweaked by vendors to attract buyers' attention is the title (Belch and Belch, 2003; Pieters et al., 2010).

Titles not only inform readers in general about the content of an advertisement, they also encourage them to read further (Dor, 2003; Ifantidou, 2009). Just like clickbait titles persuade readers to click on links by using misleading or exaggerated headlines (Pengnate, 2019), vendors could lure buyers by making the appearance, rather than the content, of their advertisement attractive.

One way to attract attention through appearance is by creating visual contrast (Narayana and Markin, 1975; Solomon et al., 2014). For example, Davies (1987) suggested that brand names with innovative spelling are generally used to attract readers' attention “simply by the virtue of its being different and unexpected” (p. 48). In addition, research on regular advertisement demonstrated that a high visual contrast between pictures of brand names and background pictures could make it easier to identify and recognise brands, and improve ad comprehensibility (Pieters et al., 2010). Another way to visually attract attention is by simply increasing the text size. Pieters and Wedel (2004) found that an increased text size in licit advertisements increased both the likelihood that an advertisement was viewed and the time spent looking at the advertisement.

Attracting attention through visuals of advertisement titles on illicit online markets might be done by creating contrast with other titles or by increasing the text size of the title. This contrast could be created by modifying the length of the title or varying the use of capitalisations or symbols. In addition, although the font size of advertisements could not be altered, vendors could increase the visual size of the title by creating longer titles or using more capital letters. To test to what extent title intensity affects criminal consumers' behaviour, we formulate the following hypotheses:

*H5: Titles containing either very few or a lot of capital letters generate higher increases in view count than titles that do not contain very few or a lot of capital letters*.

*H6: Titles that are very short or very long generate higher increases in view count than titles that are not very short or very long*.

## 3 Materials and methods

In the current study, we aim to examine the effect of the asking price, social endorsement cues and title characteristics on increases in advertisement view counts. To do so, we collected advertisements on an illicit online market accessible through the Tor network. We selected one market from all darkweb markets listed on the dark.fail website in July 2019 where account credentials were sold. Dark.fail was a well-known source listing popular hidden services, such as darkweb markets and forums.

**Figure 1:**
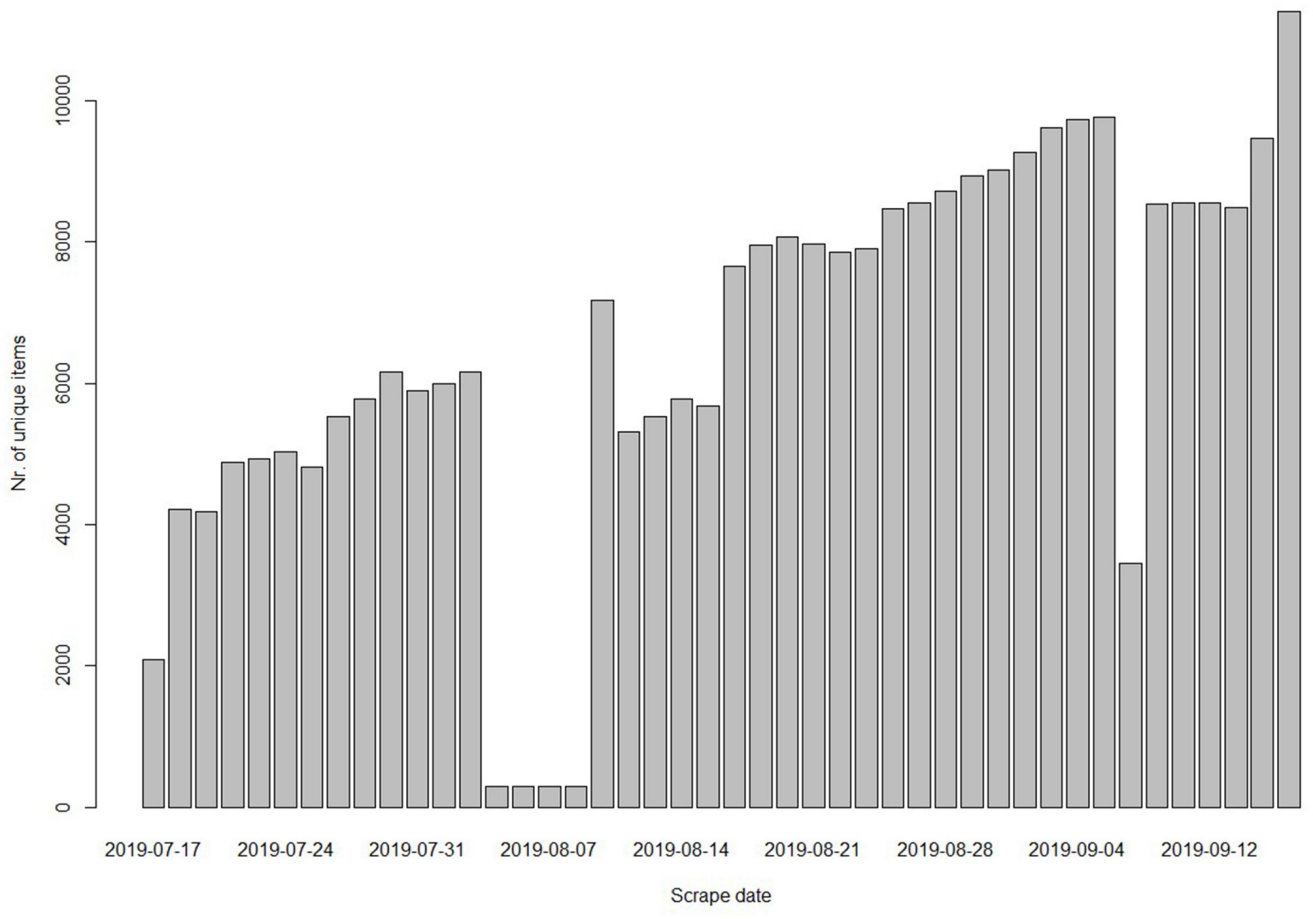
The number of ads scraped each scrape day.

To select a market for analysis, we applied two main criteria. First, we only considered English-language markets. Second, for each advertisement, all required information for our analyses (i.e., the number of views, asking price, post date, vendor status, title, and vendor's nickname) had to be provided on the overview page of the market.^1^ Only a few markets met these criteria. To select our final market for analysis, we examined which market appeared to have the most ads relating to stolen account credentials. To apply this final selection criterium, we examined the number of advertisements in the product categories as created by the markets. Generally, online marketplaces cluster different types of products into main and subcategories. Stolen account data were frequently placed in a main category called “Fraud.” Therefore, we finally selected the market with the highest number of ads in the Fraud category at the time of data collection, which was Apollon Market. Because we noted that relevant ads were placed in other categories as well, we decided to scrape all overview pages on the market.^2^ In total, we scraped 14,337 unique ads for 2 months, starting mid-July up to mid-September 2019. Figure 1 visualises de number of ads scraped each day the scraper was running. Note that we did not run the scraper in the weekends and that there were five days when the structure of the market changed so that our scraper could not scrape all advertisements during those days. In the next paragraph, we outline how we cleaned our dataset and selected relevant ads on account credentials for analysis.

### 3.1 Data cleaning and selection

Our data cleaning process involved several steps of removing data. When scraping our data, sometimes the same ad was scraped twice on the same day even though the scraper ran only once a day. This duplication occurred probably because ads were continuously added which in turn caused the ads to move between overview pages. These duplicates were removed from our dataset by keeping only the first observation of each ad on each day. We also removed ads that were posted before our first scraping date so we could analyse the increases in views from the day the ads were posted. As explained in the next section, we use the number of views as a proxy for advertisement popularity.

**Figure 2:**
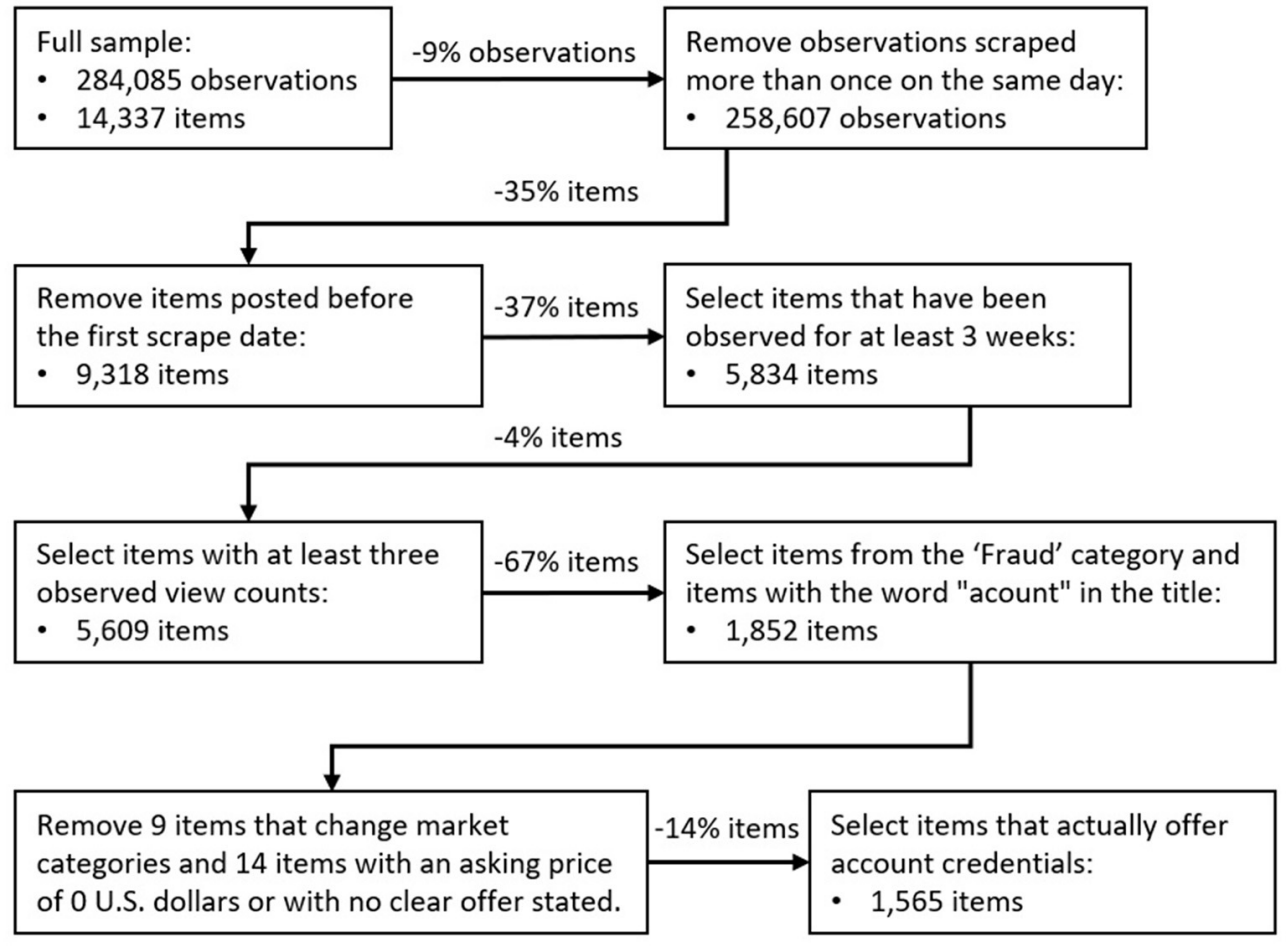
Data selection flow.

When cleaning our data, we noticed that several advertisements were not scraped even though the scraper was running and functioning well. We assume that these advertisements were then hidden from view by their vendors. This hiding resulted in additional missing observations and thus missing views. We dealt with these missing observations in two ways. First, we decided to impute daily views. This process is explained later. Second, we conducted analyses with both daily and weekly views. Our dataset with daily views contains more observations, but because weekends were never scraped, the data set with weekly views contained less imputed, and thus less “fabricated”, data. Because a panel data analysis requires changes in the dependent variable over time, we selected ads that were observed for at least 3 weeks and had at least three observed views. Our data cleaning and selection process is illustrated in Figure 2.

**Figure 3:**
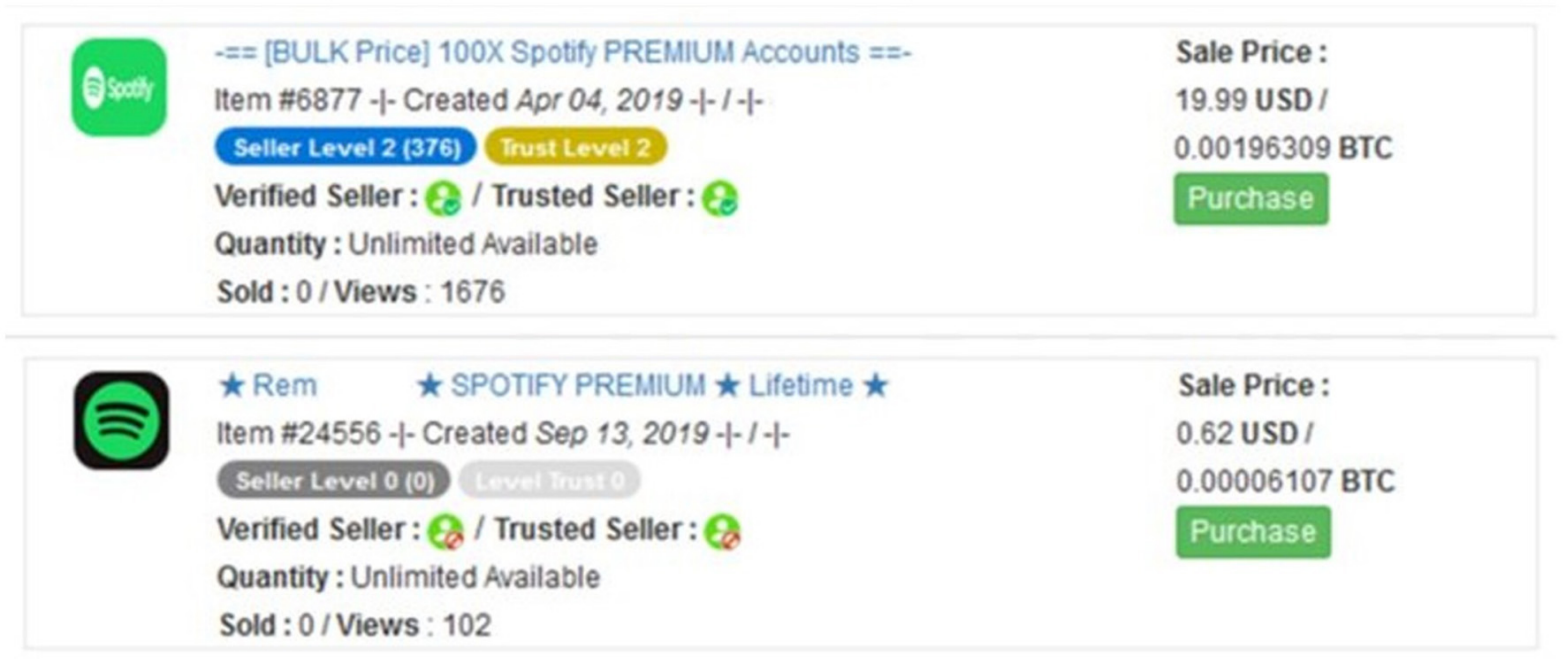
Two advertisements offering accounts.

To select relevant advertisements, we distilled ads offering account credentials. Therefore, we selected all ads containing the word “account,” irrespective of the use of capital letters as well as ads from the market main category “Fraud.” This category contained the subcategories “Accounts & Bank drops” and “Other” which both were seen to contain advertisements offering account credentials. Figure 3 illustrates two typical ads offering account credentials. To refine our data sample, we manually coded the product and account type actually on offer by reading the ad titles. Sometimes ad titles included the word “account” but offered, for instance, tutorials on how to hack accounts or hacking as a service (Mirian et al., 2019). During this manual coding, we also noted that some ads had an asking price of 0 U.S. dollars. Ads not offering accounts or without an asking price were removed from the sample as well. Eventually, the final sample of ads only offering accounts totalled 1,565 unique ads posted by 20 different vendors.

Table 1 presents an overview of the number of ads of different account types that were posted in different market categories. “Finance” account types include, for instance, bank accounts and cryptocurrency accounts. “Entertainment” account types include mainly video and music streaming accounts. “Other” account types include, amongst others, web shop accounts and social media accounts. “Porn” and “VPN” account types include accounts offering memberships of porn channels and VPN services. Other main market categories than “Fraud” containing advertised accounts are, “Carded Items,” “Hosting & Security,” “Digital Goods,” “Services” and “Other.”

**Table 1:** Number of advertisements by account type in the Fraud and other market categories.

	**Finance**	**Entertainment**	**Other**	**Porn**	**VPN**
Fraud	85	54	42	206	9
Other	4	150	31	940	44
Total	89	204	73	1,146	53

### 3.2 Variable selection

#### 3.2.1 Dependent variable

We use the number of advertisement views as a measure of ad popularity. This view count represents how many times the advertisement was clicked on. The view count shown for each ad on the website is the cumulative number of views the ad had received since it was posted. Because of the large market size and the expected presence of scrapers that either click all ads or randomly click ads to gather information on the market, we assume the cumulative view count to increase every day for all ads. Nevertheless, the rate at which these views increase, especially after a change in the explanatory variables, distinguishes the more popular ads from the less popular ads. Therefore, we use the *increase* in views as our dependent variable. We obtained the increases in views by subtracting the views at a specific moment in time (*t*) from the next moment in time (*t* + *1*).

Because the scraper did not run during the weekends, we chose to impute daily views and analyse both daily (M = 15.31; SD = 10.94; median = 14) and weekly (M = 116.30; SD = 71.50; median = 100) increases in views. The sample of weekly views was generated by analysing only the values observed on a specific weekday; here we randomly chose Wednesday. Note that a number of ads were never observed on Wednesday, so this sample only contained 1,128 ads. The ads that were not included in this sample mainly offered credentials for porn (*n* = 365) and entertainment (*n* = 60) accounts.

To impute missing views, we copied previously observed views and deployed linear interpolation. A missing view was assigned the value of the previous day if the scraper was running on the day of the missing observation. Linear interpolation was performed for days when the scraper was not running. Because our data was highly skewed, we applied a square root transformation to reduce the influence of extreme values on our analyses. This transformation was applied to both daily and weekly increases in views.

#### 3.2.2 Independent variables

Our main explanatory variables are the price per item in U.S. dollars, vendors' reputation rankings, the number of sales and cumulative views, and title characteristics, namely the title length, the percentage of capital letters in the title and special title words. The price in U.S. dollars as stated in the advertisement is not always the price per item because vendors sometimes provide package deals (i.e., selling information on multiple accounts as illustrated in Figure 2). Therefore, to test the effect of price on views, we manually checked each title for the amount of items sold per ad and subsequently converted the asking price to the price per item. When quantities were not explicitly stated, we assumed the asking price to be the price per item. The price per item varied between 1 cent and 3,877 U.S. dollars, with a mean of 13.57 U.S. dollars and a median of 6 U.S. dollars.

To test our hypothesis on the effect of social endorsement of vendors, we included vendors' reputations as independent variables. Each ad displayed four types of vendor rankings. Two types were controlled by the administrators or moderators of the market, namely if a vendor was trusted by the admins or moderators and if a vendor was verified by them (Holt and Lampke, 2010; Hutchings and Holt, 2015). The other two types, so-called “Seller Level” and “Trust Level,” related to, respectively, the total number of completed sales by the vendor and the ratings provided by customers of the vendor. We included these rankings by adding them up so we could measure their combined effect. We name the rankings related to trust and verification by admins or moderators “market endorsement,” while the rankings related to buyer experiences “buyer endorsement.” The market endorsement variable had three levels: (1) not trusted or verified, (2) either trusted or verified, and (3) both trusted and verified. The first level was observed 711 times, the second level 1,882 times, and the third level 30,629 times. The buyer endorsement variable simply summed up the two buyer endorsement rankings resulting in a scale ranging from 0 to 4. Because ranking level “3” occurred only nine times in our sample, we merged this with ranking level “4.” After merging, the ranking levels 0, 1, 2, and 3/4 were observed, respectively, 30,151, 565, 2,157, and 350 times.

To test our hypothesis on the effect of social endorsement of products, we included the number of sales and cumulative views as independent variables. The sales number represents how often the advertised item already had been sold. Sales imply that others found the ad interesting and the vendor trustworthy enough to complete a financial transaction. In a similar fashion, cumulative views signal previous interest from potential buyers. Cumulative sales counts ranged from 0 to 37, while cumulative view counts ranged from 0 to 4,233.

Finally, to test our hypotheses on the effect of attention, we computed for each title the length and the percentage of capital letters it contained. The percentage of capital letters related to the portion of letters in the title, i.e., regardless of how many numbers and symbols the title contained. We intended to test the effect of symbols as well, but the number and type of symbols varied too little between titles to make meaningful predictions with this variable. After computing the title length (*M* = 47.16; *SD* = 7.51; median = 48) and percentage of capital letters (*M* = 47.22; *SD* = 33.98; median = 49), we calculated the deciles of these variables to measure the effect of deviation from the general appearance of titles. These deciles are included in our analyses. When coding the ads by hand, we also noted that vendors sometimes include special words in their ad titles, presumably to attract additional attention. Therefore, we marked titles when these included one of the following words: “premium,” “free,” “fresh” or “sale.” The words “premium” and “free” occurred in 1,104 and 48 titles, respectively, while the words “fresh” and “sale” only occurred in 19 and 2 titles, respectively. Therefore, we only include the presence of the words “premium” and “free” in our analyses.

#### 3.2.3 Control variables

Besides the main predictors of interest, we also include several control variables in our statistical analyses. These control variables relate to the type of items advertised, the presence of duplicate items on the market and the market size. Previous research demonstrated that prices could differ greatly between different types of accounts (Holt and Lampke, 2010; Madarie et al., 2019). In addition, some account types could be more interesting to potential buyers than other types because, for instance, they are easier to monetise. Although the market provided predefined product categories for vendors to place their ads in, we only include the manually coded variable of account type. This variable provides a more accurate measure of the type of product advertised since vendors did not always place their advertised products in the corresponding market categories.

While manually coding account types and individual item prices, we noted that several vendors placed advertisements with the same title multiple times on the market. We consider these ads as duplicates. These duplicates were sometimes placed in different main categories or subcategories. Because duplicates in the same subcategory (n_ads_ = 177) were most likely to be seen more than once by potential buyers, we included a duplicate variable in our analysis where 0 = not duplicated and 1 = duplicated.

Finally, we included a market size variable because the number of ads we scraped steadily increased over time which could also affect how often ads are viewed. This market size variable thus represents the number of ads present on the market each day. However, on four consecutive days in August and 1 day in September, the number of scraped ads was notably lower due to changes in the market structure as explained before (see Figure 1). We filled these gaps by calculating the increase in ads between the “normal” days and then incrementally adding the average increase starting from the last day that was correctly scraped.

### 3.3 Model specification

To predict the effects of price, endorsement signals, and attention attributes of titles on increases in ad views, we employ a panel design (Halaby, 2004). With this method, we could analyse variation *between* advertisements as well as *within* advertisements. Two commonly applied panel designs are fixed and random effects models. With fixed effects (FE) models, one could analyse the variation within items over time. FE models have the important advantage that they do not assume exogeneity, i.e., independence between the predictor variables and unobserved heterogeneity. However, fixed effects models eliminate *all* time constant unit heterogeneity, i.e., all variation caused by both unmeasured and measured time constant variables. For our study, this implies that we do not have to worry about the potential effects of unmeasured time-constant vendor characteristics, but that we are also unable to estimate the effect of time-constant title elements that we did measure.

One way to overcome this problem is by estimating a random effects (RE) model. RE models also allow for estimation of the effects of time constant predictors. However, to obtain unbiased and consistent estimators, RE models hinge on several assumptions. One of the assumptions frequently argued to invalidate RE models is the exogeneity assumption (Brüderl, 2005; Ousey et al., 2008). A significant Hausman test suggested that this assumption was not met which implies that an RE model was not suitable for our data.

As a solution, we estimated a “hybrid” model, also known as a “within-between” model, which combines the strengths of both FE and RE models by estimating the effects of both time-varying and time-constant predictors (Halaby, 2004; Schunck, 2013; Long, 2023). In addition, it includes a vector which represents the means of each time-varying variable. This vector acts as an instrument to account for the correlation between time constant predictors and random variables which is based on between-unit variation (Halaby, 2004). In other words, it corrects the estimates of time-constant predictors for between-cluster differences (Schunck, 2013). All of our analyses were conducted in R.

#### 3.3.1 Time-constant and time-varying predictors

To determine which variables to include as time-varying or time-constant in the main analysis, we checked how often the values of the independent variables changed. As demonstrated in Table 2, nearly half of the vendors (*n* = 11) in our sample changed the prices of their ads. In total, the prices of 68 ads changed during our scraping period with more prices going down rather than up.

**Table 2:** Frequency of daily changes in independent variable values over time.

	**n**_**change**_		**n_ads_**	**n_vendors_**
	**Down**	**Up**	
Price (USD)	459	297	68	11
Buyer endorsement	30	449	405	7
Market endorsement	0		0	0
Sales	N/A	187	116	13
Cumulative views	N/A	31,653	1,565	20
Titles	18		14	4
Title: caps deciles	0		0	0
Title: length deciles	3	3	6	2
Title: “premium”	0		0	0
Title: “free”	0		0	0

Furthermore, although buyer endorsement usually increased, it decreased 30 times which implies that vendors could actually suffer from negative feedback provided by buyers. Market endorsement never changed in this sample.

As for the number of sales and cumulative views, it appeared that only a small number of ads generated actual sales, namely seven percent (*n*_*ads*_ = 116). Conversely, all ads generated views every day except for four ads. However, the view count of each of these four ads only stagnated once during the scraping period.

Finally, the titles of fourteen ads changed a total of eighteen times. Upon closer look, these changes implied, for instance, a few additional exclamation marks or a change in stated asking price. The title length deciles in which these ads fall merely changed six times. The deciles relating to the percentage of capital letters did not change over time, nor did the presence of special words.

These statistics imply that nearly all independent variables can be treated as time-varying predictors, whereas market endorsement and title characteristics should be treated as time-constant predictors. Even though two vendors changed their titles to the extent that these subsequently fell into a decile or became more “normal,” these changes occurred too little in the full sample to be useful as a time-varying predictor. As for the control variables, the account type, the assigned market category in which the ads were placed, and duplicates are time-constant predictors as well. On the contrary, the market size, unsurprisingly, increased daily and is thus treated as a time-varying predictor.

## 4 Results

### 4.1 Descriptive statistics

In this results section, we first discuss several descriptive statistics to provide a deeper understanding of our data before we discuss our panel data models. Although this paper focuses on factors affecting advertisement view counts, we also relate these factors to sales counts. As previously explained, sales counts represent how many sales the ad generated. However, because we were unable to collect data on the full sales process, we cannot analyse sales counts as a dependent variable beyond these initial descriptive statistics. In this section, we first discuss notable patterns relating to our categorical predictor variables and then examine bivariate correlations between several continuous variables. Although we tested several patterns for significance, even the weakest relationships turned out to be significant due to our large sample size. Therefore, we only highlight relationships that directly relate to our hypotheses and correlations stronger than 0.15.

Although our hypotheses relate to advertised credentials in general, we suspected that different types of credentials could attract buyers with different interests. Therefore, we first demonstrate how the increase in views and the number of sales vary along different types of accounts on offer. Subsequently, we provide several statistics relating to endorsement levels and title characteristics. Finally, we present correlations between our continuous variables before proceeding to the discussion of our panel data models.

A comparison of increases in views for different account types and main categories is provided in Table 3. A one-way ANOVA test demonstrated significant differences in increase in views between the different types of accounts on offer (*F*_(4)_ = 464.50, *p* < 0.01). Advertised finance and VPN accounts appear to have higher increases in views on average than the other advertised account types. Interestingly, when considering the average number of actual sales by account type over time, the differences between account types seem more pronounced. Again, finance accounts and VPN accounts appear to have higher sale counts than the other advertised account types.

**Table 3:** Mean (SD) of increase in views and total sale count by account type.

	**Finance**	**Entertainment**	**Other**	**Porn**	**VPN**
Increase views	18.63 (13.37)	14.51 (7.00)	15.35 (6.43)	14.60 (9.66)	27.37 (27.02)
Sales	1.07 (4.00)	0.05 (0.24)	0.14 (0.47)	0.03 (0.18)	0.21 (0.72)

When considering the endorsement variables (see Table 4), the differences in increases in views were significant again between both buyer endorsement levels (*F*_(3)_ = 32.87, *p* < 0.01) and market endorsement levels (*F*_(2)_ = 62.35, *p* < 0.01). However, although the increase in view count appears to slightly decline with market endorsement levels, there appears to be no consistent increase or decline with buyer endorsement levels. As with the different advertised account types, we noted again more pronounced differences in the actual number of sales. Upon closer look, these sales counts appeared to vary more with account type and less with endorsement levels. Interestingly, we also noted that the asking price per item was negatively related to the level of both buyer and market endorsement. In other words, the higher the endorsement level of a buyer, the lower the asking price appeared to be. Finally, when considering a possible relationship between market and buyer endorsement, we did not notice clear patterns indicating a positive or negative relationship between market endorsement levels and buyer endorsement levels.

**Table 4:** Mean (SD) of increase in views and several predictor variables by endorsement levels.

	**Buyer endorsement**				**Market endorsement**		
**Level**	**0**	**1**	**2**	**3**+	**0**	**1**	**2**
Increase views	15.36 (11.01)	17.81 (6.82)	13.54 (10.09)	17.32 (13.28)	19.05 (10.30)	16.76 (12.21)	15.13 (10.84)
Sales	0.04 (0.25)	0.83 (1.66)	0.23 (1.06)	3.19 (8.03)	0.57 (1.33)	0.71 (3.77)	0.05 (0.33)
Price	12.06 (100.85)	17.41 (17.34)	6.22 (5.32)	2.22 (3.27)	163.37 (632.71)	22.02 (44.62)	7.51 (7.99)
Most offered account type	Porn	Finance	Porn	Finance	Finance	Finance/enter-tainment	Porn

As for the relationship between title characteristics and the increase in views, titles with mainly capital letters appeared to attract more views than less capitalised titles. Furthermore, a one-way ANOVA demonstrated that titles containing the word “free,” presumably offering products for free, generate less sales than titles not containing that word (*F*_(1)_ = 4.92, *p* < 0.05). Conversely, titles containing the word “premium” generated more sales than titles not containing that word (*F*_(1)_ = 57.71, *p* < 0.01).

Table 5 presents bivariate correlations between the continuous predictor variables and the increase in views. Notably, the increase in views appeared to be weakly negatively related to ageing ads and market size growth. In other words, the older ads become and the more ads are posted on the market, the less views each ad gets. However, the more cumulative views an ad has generated, the more subsequent views it appears to attract. Furthermore, the number of cumulative views also appears to relate weakly to the number of cumulative sales. Unsurprisingly, the age of an ad is positively related to the cumulative views it has generated and to the market size. Interestingly, the asking price does not appear to share any notable relationship with any of the other variables in Table 5.

**Table 5:** Correlations between increase in views and continuous predictor and control variables.

	**Increase in views**	**Price**	**Sales**	**Cumulative views**	**Age**
Price	0.01				
Sales	0.08	−0.01			
Cumulative views	0.17	−0.02	0.20		
Age	-0.17	−0.04	0.06	0.62	
Market size	-0.18	0.02	0.02	0.34	0.67

### 4.2 Panel data models

Finally, we estimate three linear panel data models to predict the effect of several ad characteristics on the popularity of ads as measured by the increase in daily as well as weekly increases in views. As outlined in the previous sections, we include the asking price, buyer and market endorsement cues, cumulative views and sales, and title characteristics as independent variables. Furthermore, we include the account types on offer, whether or not an ad is posted multiple times, and the market size as control variables in our models. The asking price, cumulative sales and views, and the market size are mean-centred. Advertisement age is included in the models as the time or wave component while the different ads constitute the different panels. The results of our models are presented in Table 6.

**Table 6:** Panel model results for the square root of increases in views.

		**FE model**	**Hybrid model 1**	**Hybrid model 2**
		**Daily view increase**		**Weekly view increase**
	**Predictors** ^a^	*β **(SE)***	*β **(SE)***	*β **(SE)***
Within effects	Price	−0.64 (0.63)	−0.48 (1.07)	−1.91 (3.17)
	Buyer endorsement	0.02 (0.02)	0.04 (0.01)^**^	0.27 (0.03)^**^
	Sales	−0.16 (0.08)^*^	−0.13 (0.02)^**^	−0.34 (0.06)^**^
	Cumulative views	−0.30 (0.08)^**^	−0.12 (0.02)^**^	−0.07 (0.04)
	Market size	−0.06 (0.03)	−0.13 (0.01)^**^	0.01 (0.02)
Mean time-varying predictors	Price		−0.01 (0.01)	−0.01 (0.02)
	Buyer endorsement		0.10 (0.02)^**^	−0.17 (0.05)^**^
	Sales		−0.01 (0.01)	0.02(0.02)
	Cumulative views		0.73 (0.01)^**^	0.32 (0.02)^**^
	Market size		0.55 (0.02)^**^	0.18 (0.05)^**^
Time-constant predictors	Accounts: other		0.04 (0.03)	0.12 (0.07)
	Accounts: entertainment		−0.00 (0.02)	−0.01 (0.05)
	Accounts: VPN		0.22 (0.03)^**^	0.42 (0.08)^**^
	Accounts: finance		0.27 (0.04)^**^	0.60 (0.10)^**^
	Duplicate		0.01 (0.02)	0.15 (0.04)^**^
	Title capital deciles: few		−0.03 (0.02)	−0.02 (0.04)
	Title capital deciles: a lot		−0.04 (0.02)^*^	0.08 (0.05)
	Title length deciles: short		0.02 (0.02)	−0.00 (0.05)
	Title length deciles: long		0.05 (0.02)^**^	0.01 (0.04)
	Title words: “free”		−0.13 (0.05)^*^	-
	Title words: “premium”		0.07 (0.01)^**^	0.10 (0.03)^*^
	Market endorsement: 1		−0.53 (0.05)^**^	0.42 (0.14)^**^
	Market endorsement: 2		−0.54 (0.05)^**^	−0.0 (0.13)

^*a*^The reference category for market endorsement was the lowest rank, i.e., level “0.” The reference category for accounts was the largest category, namely porn accounts. The reference category for duplicates was the non-duplicate category. The reference category for the title characteristics were “normal” titles, i.e., titles without characteristics of the highest and lowest deciles, and titles without the words “free” or “premium.” ^*^p < 0.05, ^**^p < 0.01.

We first estimated an FE model with only time varying predictor variables. The Breusch-Pagan test was significant for these models which means heteroskedasticity was detected. Therefore, we applied a robust covariance matrix to account for heteroskedasticity. According to this first model (*R*^2^ = 0.07, adjusted *R*^2^ = 0.03), neither the asking price nor buyer endorsement levels appear to influence the increase in views. Furthermore, the cumulative sale and view count appeared to be negatively related to the increase in views. This implies that the more sales or views an advertisement has generated, the less views it subsequently receives.

However, as stated before, we are also interested in the effect of time-constant title characteristics and market endorsement levels on ad popularity. To this end, we estimated a linear hybrid model predicting the increase in daily views. When plotting the data, we noted that ads showed diverging increases in their view counts over time. Therefore, we plotted a hybrid model without individual slopes (*BIC* = 88,204) and a hybrid model with individual slopes (*BIC* = 87,257). Because these models are highly similar, as was expected given the highly similar BIC-values, we only present the model with individual slopes in Table 6.

In this first hybrid model, buyer endorsement does appear to affect the increase in daily views. More specifically, the higher vendors' ranking, the more views their advertisements generate. In addition, the larger the market size (i.e., the more ads offering account credentials are posted on the market), the less views each ad generates. Furthermore, similar to the FE model, an increase in cumulative sales and views count relates to a lower increase in subsequent views.

The additional results for time-constant predictors confirm our previous descriptive results relating to the different types of accounts. In this model, VPN accounts and financial accounts generate more views than porn accounts, whereas entertainment and other types of accounts generate similar view counts. Furthermore, the way a title looks appears to affect increases in views as well. Titles containing mostly capital letters generate less views, while very long titles generate more views when compared to normal titles. In addition, special words in the title also affect the increase in views. The word “free” is associated with lower increases in views whereas “premium” leads to a higher increase in views. The results for time-constant vendor ranking coefficients are mixed. Whereas, higher average buyer endorsement levels are related to higher increases in views, which is similar to the FE coefficients, higher average market endorsement levels are related to lower increases in views.

At last, we estimated a hybrid model allowing for individual slopes (*BIC* = 12,516) with the *weekly* increases in views. The results of this analysis are demonstrated in Table 6 as well. As in the previous models, the asking price does not affect increases in views. Furthermore, buyer endorsement is again positively related to the increase in views and sales is negatively related to the increase in views. The cumulative views and market size were not related to increases in views in this model.

The results for time-constant predictors are similar to the previous model when considering account type. Again, VPN account and financial accounts generate more views than porn accounts. However, according to this model, title appearance does not affect increases in views. The word “premium” remained positively associated with a higher increase in views. Due to the lower number of titles containing the word “free” (*n* = 19), we did not include this variable in this model. Finally, as with the daily model, the results for time-constant vendor endorsement variables are mixed. Average levels of buyer endorsement were related to lower increases in views, whereas only the moderate level of market endorsement (i.e., either “trusted” or “verified”) was related to higher increases in views. Vendors who were both trusted and verified did not generate more ad views than vendors who had neither a trusted nor verified status. Finally, in both models for daily and weekly views, higher average cumulative view counts and market size were related to higher increases in views.

## 5 Discussion

To study illicit online market dynamics, we broadened our theoretical approach by departing from knowledge on licit consumer behaviours. This enabled us to examine factors affecting criminal consumption that have been neglected by criminologists in previous studies on offender decision-making. Specifically, we analysed the effects of price, social endorsements and title elements on the popularity of darkweb market advertisements offering account credentials. To this end, we estimated several panel data models. These models reveal counter-intuitive results. In this section we relate these results to our hypotheses, discuss several limitations of this study and provide suggestions for future experimental studies to improve our knowledge on the trade in stolen credentials. This knowledge could inform strategies to disrupt online criminal businesses, such as diverting attention away from real illicit vendors or diminishing the attractiveness of the products they offer.

As for our first hypothesis relating to the asking price, surprisingly, we found no effect on the popularity of advertisements. Neither the stated asking price nor the converted price per item affected the increase in advertisement views. This is surprising because the price is clearly stated on the advertisements overview pages (see Figure 3) and one of the few measures that can be used by potential buyers to make an informed rational decision. This finding not only contradicts expectations derived from a utilitarian perspective, but also previous research demonstrating that higher prices relate to less sales of drugs items on illicit online markets (Przepiorka et al., 2017; Norbutas et al., 2020).

A possible explanation for this contradictory finding is that credentials are not regular products of which the value can be easily calculated and compared. Account credentials are most useful, and thus most valuable, when they have not been exploited by others. If they have been exploited by others, then it is more likely that monitoring systems have detected malicious activity and prompted security measures against further abuse (Shulman, 2010). Whether or not exploitation by others has occurred is hard to determine by price. In fact, this is hard to determine at all for potential buyers. Moreover, one could even argue that not even vendors can always be certain that the credentials they sell actually work, since account users could change their passwords at any given time. Due to this uncertainty, advertised account credentials are arguably high-risk products. As suggested by other researchers, endorsements by others are particularly important for products where the actual utility is uncertain because this can only be established after purchasing the product (Dellarocas, 2003; Metzger and Flanagin, 2013; Chen et al., 2021). Therefore, we tested if vendor status related positively to the popularity of ads offering account credentials.

Our second, third and fourth hypotheses relate to these endorsement cues. Our second hypothesis stated that vendor status relates positively to ad popularity. The fixed effects in our hybrid models suggest that buyer endorsement is indeed related to higher ad popularity, which confirms previous research on both licit (e.g., Serra Cantallops and Salvi, 2014) and illicit (Przepiorka et al., 2017) online markets. However, the time-constant predictors relating to vendor endorsement reveal mixed results. We suspect this might be due to the small number of vendors in our sample and elaborate on this limitation in the next section.

Our third and fourth hypotheses stated that the cumulative number of sales and views, respectively, also relate positively to ad popularity as these could be interpreted as product endorsement signals from previous buyers. To this end, we examined the effects of both the cumulative number of sales and views on ad popularity. Surprisingly, we found that potential buyers are less interested in advertised accounts when others have shown interest in these items by either clicking on the ad or actually buying advertised credentials. As explained before, this makes sense if account credentials are considered high-risk products. If more people demonstrated interest in certain credentials or have actually bought these credentials, it is more likely that these credentials have been exploited by others. This in turn increases the chance that security measures have been triggered, which could render the credentials useless.

Our final hypotheses relate to the visual appearance of titles. Here we noted a few differences between our daily and weekly models. Whereas, particularly long titles and the use of a lot of capital letters appeared to affect ad popularity in our daily hybrid model, the visual appearance did not seem to relate to ad popularity in our weekly hybrid model. However, the use of the word “premium” was consistently positively related to ad popularity in both models. This could be explained by the notion that “premium” accounts are usually accounts with more user options. For instance, premium entertainment accounts could allow users to stream videos on more devices than regular accounts. It therefore makes sense that these ads attract more views than ads not offering premium accounts. In addition, the use of the word “free” in a title appears to decrease ad popularity, which is in line with our argument that the more people could use certain credentials, the less valuable these credentials are.

### 5.1 Limitations and suggestions for future directions

While our study offers valuable insights into consumer behaviour on illicit online markets, it is important to recognise its limitations. One limitation lies in the relatively small number of vendors included in our dataset. Despite the extensive collection of advertisements over an extended period, our dataset includes a limited number of vendors. This limitation may contribute to the discrepancies observed in our findings relating to the time-constant mean values of the vendor rankings. It is worth noting that the vendors in our sample were not chosen based on specific vendor characteristics. However, it is possible that these vendors differ in some aspects from those excluded during our data selection process. To strengthen the validity of our findings, future research could consider expanding the dataset by including a larger number of vendors. This can be achieved by either scraping additional advertisements or employing alternative selection criteria.

Furthermore, it would have been intriguing to explore the factors influencing actual sales. Unfortunately, our data collection did not extend to scraping the actual advertisement pages. This limited our access to the full advertisements, including information on the sales process, such as warranties and preferred payment methods stated by the vendor, which could also affect purchase decisions. Similarly, we were unable to analyse reviews from previous buyers displayed on these advertisement pages. Existing research on licit online markets, exemplified by Serra Cantallops and Salvi's (2014) literature review, underscores the significant impact of customer reviews on product valuation and purchase decisions. Examining these reviews and their influence on actual sales could offer valuable insights for cybersecurity practises aimed at deterring the sale of stolen credentials.

While panel data analyses offer valuable insights into causal effects, true causal relationships between ad characteristics and ad popularity are best established with experimental studies. To this end, researchers could, for instance, pose as vendors on illicit online markets, allowing precise control over several predictor variables. For instance, researchers could post nearly identical ads while manipulating elements such as title characteristics or asking prices to gauge their impact on ad popularity and potential sales. This could experimentally validate, or disprove, the findings of this study.

In addition, researchers could also systematically vary the type of product offered. This would generate more insights into the extent to which the factors assumed to affect illicit consumer decisions shift based on the type of product in consideration. If consumers searching for different types of products are differently affected by advertisement elements, that would suggest varied strategies are necessary to deter them from purchasing illegal products online.

However, it should be noted that not all variables are easy to manipulate. For instance, the manipulation of vendor status poses challenges, given that this status often reflects actual feedback from previous customers. Several online platforms used for illicit trade do not even implement vendor ranking systems, as is the case with, for instance, the increasingly popular Telegram app (Blankers et al., 2021). Within this context, it is also challenging to determine ad views and sales due to the absence of public count metrics associated with posted ads. These challenges could be overcome as well by experimentally posting advertisements. This allows full control over ad content as well as keeping track of the replies the ads generate.

The insights generated with these studies, as well as with our own study, have both theoretical and practical implications. For instance, based on our unexpected findings, we encourage researchers studying criminal consumers and the sales of illicit products online to broaden their theoretical perspectives to achieve a more integrated understanding of illicit online market dynamics. Practically, these insights could aid in disrupting illicit online business models. For instance, cybersecurity specialists could mimic vendors by posting highly attractive advertisements, thereby diverting potential buyers' attention away from legitimate advertisements. Alternatively, if our findings hold up in other studies, cybersecurity specialists could demonstrate interest in advertised credentials, thereby making these products less attractive to potential buyers. This way, a better understanding of what factors affect criminal consumer decision-making could inform more targeted and more effective cybersecurity strategies aimed at discouraging the online sales of illicit products.

### 5.2 Conclusion

This study aimed to provide a better understanding of illicit online market dynamics by analysing the simultaneous effects of factors usually not examined by criminologists on advertisement popularity. Drawing from knowledge on licit consumer behaviour, we examined how the asking price, vendor rankings and title appearance appeared to affect illicit consumer decision-making. Our findings suggest that potential buyers of illicit products on online markets do not behave as regular online consumers. The fact that most of our hypotheses were disconfirmed could be explained by considering account credentials to be high-risk goods that, paradoxically, become less attractive when they become more popular. Overall, this study illuminates several unexpected nuances of illicit online consumer behaviour when examined through a non-traditional theoretical lens.

## Data availability statement

The datasets presented in this article are not readily available because the dataset is available by request to the Netherlands Institute for the Study of Crime and Law Enforcement. Requests to access the datasets should be directed to www.nscr.nl.

## Ethics statement

The studies involving humans were approved by the Ethics Committee for Legal and Criminological Research (CERCO). The studies were conducted in accordance with the local legislation and institutional requirements. Written informed consent for participation was not required from the participants or the participants' legal guardians/next of kin in accordance with the national legislation and institutional requirements.

## Author contributions

RM: Conceptualisation, Formal analysis, Investigation, Methodology, Writing—original draft, Writing—review & editing, Data curation. CP: Funding acquisition, Methodology, Project administration, Resources, Supervision, Writing—review & editing. MW: Writing—review & editing, Methodology, Supervision.

## Footnotes

^1^Sometimes this information was provided inside the advertisement (i.e., on the ad page rather than the overview page). Accessing that information would require us to click on the ad. However, by doing so, we would affect the view counter. To prevent this, we only considered markets that listed all this information on their overview pages.

^2^Web scraping is a programming technique with which the source code of web pages can be automatically collected and stored offline for later usage.
